# The effect of a pay-for-performance program on health-related quality of life for patients with hepatitis in Taiwan

**DOI:** 10.1186/s12955-022-02038-1

**Published:** 2022-09-05

**Authors:** Wei-Chih Su, Tsung-Tai Chen, Sien-Sing Yang, Ling-Na Shih, Chih-Kuang Liu, Chia-Chi Wang, Chien-Hsien Wu

**Affiliations:** 1 Department of Gastroenterology, Buddhist Tzu Chi Medical Foundation, Taipei Tzu-Chi Hospital, New Taipei City, Taiwan; 2 Department of Public Health, College of Medicine, Fu-Jen Catholic University, New Taipei City, Taiwan; 3 Liver Unit, Cathay General Hospital Medical Center, Taipei, Taiwan; 4 School of Medicine, College of Medicine, Fu-Jen Catholic University, New Taipei, Taiwan; 5 Lo-Sheng Sanatorium Hospital, Ministry of Health and Welfare, New Taipei City, Taiwan; 6 Department of Internal Medicine, Taipei Hospital, Ministry of Health and Welfare, New Taipei City, Taiwan; 7 Department of Urology, Fu-Jen Catholic University Hospital, New Taipei City, Taiwan; 8 Graduate Institute of Business Administration and College of Medicine, Fu-Jen, Catholic University, New Taipei City, Taiwan; 9 School of Medicine, Tzu Chi University, Hualien, Taiwan

**Keywords:** Viral hepatitis, Pay-for-performance, Regular ongoing examination, Health-related quality of life, EQ-5D

## Abstract

**Purpose:**

Chronic viral hepatitis is a major global public health problem. The guidelines suggest the long-term performance of regular ongoing liver examinations to monitor liver inflammation and screen for hepatocellular carcinoma. However, the effects of regular liver examinations on health-related quality of life (HRQoL) have not been adequately evaluated. Therefore, this study evaluated the effects of regular ongoing examinations on the quality of life of patients with hepatitis.

**Methods:**

A cross-sectional study was conducted from October to December 2016 in four hospitals in northern Taiwan. A hepatitis pay-for-performance (P4P) program was launched in 2010 to ensure that hepatitis patients have regular ongoing liver examinations. The study group consisted of patients who joined and stayed in the program for more than one year. The study assessed HRQoL utilizing the five-level version of the EuroQol-5 Dimension (EQ-5D-5L) and the EuroQoL visual analog scale (EQ-VAS). The responses for the EQ-5D-5L in hepatitis patients were transformed into the EQ-5D index according to the Taiwanese population’s value set. Sociodemographic and clinical characteristics were collected by questionnaire, and descriptive statistics were presented. A two-part model and generalized linear model with a Poisson distribution and a log link function, respectively, were used to examine the associations of the EQ-5D index and EQ-VAS score with participation in the hepatitis P4P program. We applied propensity score weighting with inverse probability weighting to control for selection bias.

**Results:**

In all, 508 patients (aged 57.6 ± 11.6 years; 60.8% male) were enrolled in this study. The mean (standard deviation, SD) reported EQ-5D index and EQ-VAS scores were 0.93 (0.12) and 75.1 (13.8), and the median (interquartile range, IQR) values were 1 (0.108) and 80 (15), respectively. The study group had a moderately significantly higher EQ-VAS score (mean ratio = 1.029, *P* < 0.001). However, the differences in the EQ-5D index scores between the study and control groups were not significant.

**Conclusion:**

Patients with hepatitis partially benefited from receiving hepatitis P4P in Taiwan, which featured regular ongoing liver examinations, in that their EQ-VAS scores were enhanced but not their EQ-5D index scores.

## Introduction

Viral hepatitis is a major global public health problem, and mortality from viral hepatitis has increased by 22% from 2000 to 2015 globally. Hepatitis B (HBV) and hepatitis C (HCV) are the major forms of viral hepatitis and are responsible for 96% of hepatitis-related mortality. In 2015, the World Health Organization (WHO) estimated that 257 million people (3.5%) were living with chronic HBV infection and 71 million people (1%) were living with chronic HCV infection [1]. Hepatitis B and hepatitis C virus infections induce liver inflammation, which is almost always mild but persistent. A person infected with HBV or HCV may exhibit minimal symptoms for up to 30 years or more before developing any clinical signs. Thus, viral hepatitis is underdiagnosed among the population due to inadequate examination and treatment of carriers [2, 3]. Without monitoring and treatment, HBV and HCV infection can lead to life-threatening consequences, including cirrhosis and hepatocellular carcinoma (HCC). Strong and effective antiviral therapy has been introduced in recent decades and has shown promising effects in handling chronic viral hepatitis. With appropriate treatment, HBV-induced hepatitis can be controlled [4], and HCV infection can be cured [5, 6]. However, there is no true cure for hepatitis B infection and reverse hepatitis-induced liver fibrosis, which may increase the risk of developing related hepatic complications. Current guidelines suggest that all patients with HBV and/or HCV infection should regularly undergo glutamic oxaloacetic transaminase (GOT)/glutamate pyruvate transaminase (GPT) tests and should have abdominal sonography at least twice yearly for hepatitis monitoring and HCC surveillance, respectively [7–12]. For a patient with HBV, and HCV, monitoring and surveillance can aid in the early detection of early-stage tumors and improve overall survival [13].

Taiwan is an endemic area for HBV infection with a prevalence of approximately 15–20% before the massive vaccination program launched in June 1984 [14]. A recent survey shows that the hepatitis B prevalence rate of the population born after 1984 is below 1%. However, the carrier rate of hepatitis B remains high, ranging from 6.7 to 10.2 percent among the population born before 1984 [15]. Furthermore, Taiwan has an estimated hepatitis C prevalence of 3.28% (1.8–5.5%), which is higher than the global rate [12]. Based on the latest data obtained for Taiwan in 2019, HCC was the 2nd leading cause of cancer death, and chronic hepatitis and cirrhosis were the 10th leading causes of all deaths [16]. However, in 2009, only 22% of Taiwanese hepatitis patients underwent the recommended guidelines of twice-yearly follow-ups [17]. To solve this issue, the Taiwan National Health Insurance Administration (NHIA) launched a hepatitis pay-for-performance (P4P) program in 2010.

The P4P program is a reimbursement model, different from fee-for-service, that provides extra financial rewards to physicians and medical institutes based on their performance on quality measures. The hepatitis P4P program in Taiwan aims to improve the quality of patient care by increasing the implementation of guidelines that suggest preventive services and regular liver examinations twice a year [18].

Some studies have presented the effect of the hepatitis P4P program as related to the patient’s utilization rate of services and clinical outcomes. The results indicate that the P4P program improves adherence to a guideline-based examination schedule; however, the change reported is minimal [19]. Furthermore, with regard to clinical outcomes, the program has failed to decrease hepatitis-related admission events and the incidence of cirrhosis in participating patients for at least three years [20]. However, whether the program has implications for patient-reported outcomes (PROs), such as quality of life (QoL), has not yet been reported.

The WHO defines QoL as an individual's perception of their position in life in the context of the culture and value systems in which they live and in relation to their goals, expectations, standards and concerns. Those aspects of self-perceived well-being that are related to or affected by the presence of disease or treatment are defined as health-related quality of life (HRQoL) [21]. The US Food and Drug Administration officially defines HRQoL as “a multidomain concept that represents the patient’s general perception of the effect of illness and treatment on physical, psychological, and social aspects of life” [22]. In a long-lasting disease course, chronic viral hepatitis not only induces clinical symptoms but also profoundly impacts patients' daily life, similar to other chronic diseases [23]. Viral hepatitis has impacts on multiple dimensions, not merely via the physical symptoms directly caused by complications. Patients often report subjective symptoms, such as fatigue, loss of appetite, depression, anxiety, low self-esteem, and other psychological problems [24–26]. Thus, awareness of the need for comprehensive HRQoL assessments in chronic hepatitis patients has increased. Some of these symptoms and perceptions of disease management in daily life cannot be evaluated merely by clinical measures, and HRQoL measures can provide a more comprehensive evaluation from the patient perspective and have been emphasized in the management of chronic diseases [27, 28].

Given that the primary activities rewarded by the hepatitis P4P program in Taiwan are regular examinations, the incentives impact continuous follow-up by physicians [17]. In practice, guidelines for chronic viral hepatitis treatment, regular liver examinations have also been suggested, in addition to medication, for hepatitis patients [7–12]. Although previous studies have demonstrated that preventive, nontherapeutic intervention improves clinical outcomes [29], few studies have explored the effects of regular and continuous examinations on QoL. Therefore, the present study evaluated the effectiveness of the hepatitis P4P program, which features regular and continuous examinations, on QoL.

## Materials and methods

The National Health Insurance Administration (NHIA) is a mandatory single-payer health insurance program in Taiwan that provides comprehensive medical care to greater than ninety-nine percent of Taiwanese residents. The NHIA provides insurance-covered liver examinations to all patients with chronic HBV and HCV infection every 6 months. However, the attendant rate of such preventive services was only 22% in 2009 [17]. In 2010, the NHIA launched a first version of the P4P program called “Reimbursement Improvement Themes for NHIA hepatitis B and C patients” to improve the care quality of viral hepatitis patients. The sixth and also the latest version of the hepatitis P4P proposal was launched in 2016, after which there have been no changes to the hepatitis P4P proposal [18]. The program aims to encourage physicians via extra financial incentives to improve the care quality for hepatitis patients by increasing the uptake rate of guideline-based preventive services for monitoring and surveillance of disease status and chronic hepatitis-related complications in outpatient settings. The hepatitis P4P program was based on the following methods: first, a patient-centered care model was created; second, patients were proactively tracked to encourage regular visits according to guideline suggestions; third, a comprehensive and continuous care model was provided; and fourth, viral hepatitis education was improved and strengthened.

This program is open to physicians specializing in gastroenterology, family medicine, or pediatrics who provide treatment for patients infected with HBV or HCV. Once they have received continuous follow-up at the same medical facility twice with a 6-month interval, patients can be enrolled in P4P by their physicians. The follow-up must include serum GOT/GPT tests and abdominal sonography. Patients participate in the program at their will. On enrollment, patients receive health education regarding viral hepatitis via the physician or disease manager. As they enroll patients, physicians become eligible for four types of extra payments per capita: a new enrollment payment (U.S.$3); a payment for subsequent follow-up every 6 months (U.S.$3); and a payment for (1) referral of a patient with a newly screen-detected hepatic tumor, for whom the tumor was diagnosed as an early-stage (The American Joint Committee on Cancer stage I or II) HCC by subsequent examinations (U.S.$17), and (2) confirmation of the referred hepatic tumor as an early-stage HCC (U.S.$17) or detecting and diagnosing a hepatic tumor as an early-stage HCC (U.S.$34). For care continuity, the patient must be registered through the NHIA website when joining the P4P and tracked and followed by the same physician or medical institute. Any patient referral must involve an online transfer procedure via the NHIA website. A patient who is lost to follow-up by his assigned physician or medical institute for more than one year is dropped from the program. If the follow-up rate among enrolled patients falls below 20%, the NHIA will cut the physician's payment the subsequent year. The enrollment rate was approximately 38% in 2016 [30]. Low enrollment rates may be due to patient-level or physician-level factors, including physician preferences and perceptions of patients with mild or non-symptomatic hepatitis, patients who forget their carrying status, and insufficient patient education [20, 30].

### Study sample

This cross-sectional study was performed from October to December 2016, during which we collected data from patient surveys and medical record reviews at the gastroenterology clinics of four hospitals in northern Taiwan. Inclusion criteria were as follows: (1) patients infected with chronic hepatitis B and/or hepatitis C, and (2) patients aged 18 years or older. A previous study revealed that the duration of enrollment in the P4P program was associated with patient outcomes [31], indicating there may be a dose–response relationship by which QoL was superior for patients in the P4P group (more than 2 examinations) compared to patients in the control group (only one/two examinations). Therefore, we further divided all the study subjects into two groups: the P4P group (study group) and the non-P4P group (control group). Our P4P group consisted of patients who, having provided signed consent, participated in the program consecutively for more than one year.

### Patient characteristics

We collected patient sociodemographic characteristics, including age, sex, income (monthly income including subsidies from family and government < 1000 USD, 1000–2000 USD, 2001–3333 USD, and > 3333 USD), education attainment (below elementary including illiterate, junior high, college, senior high including vocational high, Bachelor’s degree, Master’s degree and above), and marriage status (married, unmarried, widow, others including separated, divorced, etc.). The following clinical characteristics were recorded: nonhepatitis-related comorbidities diagnosed by a doctor, hepatitis complications (history of hepatitis acute exacerbation, liver cirrhosis), admission or emergency department visits related to viral hepatitis within the previous year, and type of viral hepatitis (HBV, HCV or HBV/HCV coinfection). The list of comorbidities in our survey questionnaire was obtained from the Elixhauser Comorbidity Index [32].

### Outcome measures and survey procedure

Regarding the outcome measures, we chose to emphasize HRQoL rather than QoL because HRQoL focuses on overall quality of life with respect to physical or mental health and can be easily used to assess the patient’s perception of illness status and treatment outcomes [33]. We adopted the EQ-5D presented by the EuroQol Group, one of the most widely used generic scales for measuring HRQoL [34] because the EQ-5D (EQ-5D-5L) has greater advantages, including high validity, simplicity and lower time requirements [35, 36], and generates a higher minimally important difference [37] and a wider range of utility scores than other health utility measures [38] (e.g., the Short-Form Health Survey). Furthermore, the Taiwanese version of the EQ-5D-5L has been tested and has demonstrated validity, reliability, and responsiveness [39, 40].

The EQ-5D essentially consists of two parts: the EQ-5D index and the EuroQoL visual analog scale (EQ-VAS). The EQ-5D index is a self-reported but objective, multidimensional health status evaluated in five parts, including mobility, self-care, usual activities, pain/discomfort, and anxiety/depression. Each dimension has five levels that roughly correspond to no, slight, moderate, severe, and extreme problems [41]. In contrast, the EQ-VAS is a more subjective health status, which records the patient’s self-rated health on a vertical VAS, with endpoints labeled ‘The best health you can imagine (100 points)’ and ‘The worst health you can imagine (0 points).’ The VAS can be used as a quantitative measure of health outcome that reflects the patient’s own judgment. Thus, the EQ-5D index introduces preferences related to social valuations regarding health status, but the EQ-VAS does not [42]. In most cases, the EQ-5D index score correlated well with the associated VAS score [43]. However, sociodemographic characteristics, such as age, education, ethnic background [44], and psychological disposition [43], can influence an individual’s EQ-VAS scores, independent of that person’s health state. Using a value-set algorithm, the scores reported for the descriptive system in the EQ-5D can be transformed to a single measure called the EQ-5D index. This study adopted the Taiwanese value-set algorithm recently generated for the EQ-5D index [45].

When they came in for routine follow-up for chronic viral hepatitis, the study subjects were screened and selected by trained interviewers, who briefly introduced the study, obtained informed consent, and distributed self-writing questionnaires. If the respondents could not read or appeared unable to understand the questions, the interviewers were asked to read the entire questionnaire to the interviewees to ensure their comprehension. The EQ-5D was generally completed by patients in the P4P group after finishing at least the two times of regular examinations, because they were required to have participated in the program consecutively for more than one year. This research was approved by the ethics committee of the Institutional Review Board (IRB), Taipei Hospital, Ministry of Health and Welfare (IRB number IRC-IRB-4-0014-01-02).

### Statistical analysis

Descriptive statistics were used to evaluate the sociodemographic and clinical characteristics investigated. Subjects with missing values for the aforementioned variables were excluded. The variance inflation factor (VIF) was used to address the problem of multicollinearity between independent variables. A VIF above 10 indicated a high correlation, and the correspondent variable was removed [46]. Multivariable ordinary least squares (OLS) regression was applied to estimate the effects of the P4P program on the EQ-5D index and EQ-VAS score. Independent variables included age, sex, monthly income, marital status, education level, nonhepatitis-related comorbidity, hepatitis-related complications, history of recent admission or emergency department visit in the latest year because of hepatitis-related condition, and type of viral hepatitis infection, as described in the patient characteristics section. Regarding the skewness and ceiling effect of the EQ-5D index, in general, the utility values are expected to be skewed, whereby many patients would report perfect health [23, 47]. To adjust these problems, we first conducted a Kolmogorov–Smirnov test to examine normality and then used a two-part model to adjust the skewness and ceiling effect of the EQ-5D index. In general, the two parts of the model are (1) a logistic regression and (2) a generalized linear model (GLM). First, the logistic regression models the probability of disutility (1-EQ-5D index) for adjusting purposes because too many respondents report a perfect EQ-5D index. The second part of the model utilized a GLM with a gamma distribution and a log link function under the condition of probability of disutility [47, 48]. Regarding the adjustment of skewness of the EQ-VAS score, we adopted a GLM with a Poisson distribution and a log link, as reported in a previously published article [49].

To avoid selection bias regarding P4P program participation, we applied a propensity score weight (PSW) with an inverse probability weighting to weight patients in the P4P intervention and control groups [50]. Compared to the matching method, weighting the propensity score has the advantage of incorporating most of the analytical observations and can increase precision in estimating treatment effects [51]. Two of the most used propensity-score-weighting approaches are inverse probability treatment weights (IPTW) and standardized mortality ratio weights (SMRW). Inverse probability treatment weights (IPTW) involve weighting by the inverse probability of receiving the study intervention (1/propensity score for the treated group and 1/(1 - propensity score) for the reference group). Our study adopts the IPTW in propensity score weighting to balance the self-selection bias of P4P program participation. Each of the aforementioned sociodemographic (age, sex, income, marital status, education level) and clinical characteristics (comorbidity, hepatitis-related complication, admission or emergency department for hepatitis-related condition, type of hepatitis) were used to generate an inverse probability weighting (propensity score) for each patient via logistic regression, and the resulting propensity score was used in subsequent regression analysis.

All analyses utilized SAS software version 9.4 (Statistical Analysis Systems, Inc., Cary, NC, U.S.) except for the two-part model, which was analyzed by STATA 15. The results were considered statistically significant for *P* < 0.05 in all analyses.

## Results

We interviewed and collected data from 516 patients. Eight cases had missing data, seven involving income level and one involving incomplete EQ-5D scores, reducing the sample size of our study to 508. Table 1 shows the baseline characteristics of the subjects in the P4P and non-P4P groups. No significant differences were observed between the two groups in terms of clinical factors, including comorbidities or related complications. The patients in the two groups differed with regard to two demographic characteristics, namely, a high income and a high education level; there were borderline differences in terms of sex.

**Table 1:** Characteristics of study participants

	Total (*n* = 508)	P4P^a^ (*n* = 121)	Non-P4P^a^ (*n* = 387)	*P* value
Age, y (SE)	57.61 (0.51)	58.10 (0.94)	57.46 (0.61)	0.57
*Sex*				
Male	309 (60.8)	83 (69)	226 (58)	0.05
Female	199 (39.2)	38 (31)	161 (42)	
*Income*				
< U.S.$1,000	283 (55.7)	53 (44)	230 (59)	0.001
U.S.$1,000-$2,000	146 (28.7)	37 (31)	109 (28)	
U.S.$2,001-$3,333	57 (11.1)	20 (17)	37 (10)	
> U.S.$3,333	22 (4.3)	11 (9)	11 (3)	
*Education level*				
Below elementary (including illiterate)	114 (22.4)	22 (18.18)	92 (23.77)	0.02
Junior high	93 (18.3)	16 (13.22)	77 (19.90)	
College	58 (11.4)	24 (19.83)	34 (8.79)	
Senior high (including vocational high)	152 (29.9)	35 (28.93)	117 (30.23)	
Bachelor	68 (13.2)	17 (14.05)	51 (13.18)	
Master and Doctorate	23 (4.5)	7 (5.79)	16 (4.14)	
*Marital status*				
Married	380 (74.8)	102 (84.30)	278 (71.83)	0.06
Unmarried	58 (11.3)	11 (9.09)	47 (12.14)	
Widow	30 (5.9)	6 (4.96)	24 (6.20)	
Other^b^	40 (7.9)	2 (1.65)	38 (9.82)	
Any comorbidity	313 (61.6)	67 (55.37)	246 (63.57)	0.11
Any complication	86 (16.9)	22 (18.18)	59 (15.25)	0.49
Admission within one year	23 (4.5)	3 (2.48)	20 (5.17)	0.22
Emergency department visit within one year	21 (4.1)	4 (3.31)	17 (4.40)	0.60
*Viral hepatitis type*				
Hepatitis B	376 (74.0)	96 (79.34)	280 (72.35)	0.13
Other hepatitis^c^	132 (26.0)	25 (20.66)	107 (27.65)	

P4P^a^: pay-for-performance; Other^b^: separated and divorced status; Other hepatitis^c^: hepatitis C and hepatitis B/C coinfection; *SE*, standard error

Table 2 shows the distribution of EQ-5D-5L responses of chronic hepatitis patients and compares the results based on the status of P4P participation. Compared to the non-P4P group patients, patients in the P4P group displayed higher percentages in level 1 and level 2; however, we observed no significant differences between the study group and the control group in any of the EQ-5D domains.

**Table 2:** Distribution of EQ-5D-5L responses

	P4P^a^ (%)	Non-P4P^a^ (%)	*P* value^b^
	(*n* = 121)	(*n* = 387)	
*Mobility*			
Level 1	117 (97)	370 (96)	0.34
Level 2	3 (3)	14 (4)	
Level 3	0 (0)	2 (1)	
Level 4	1 (1)	0 (0)	
Level 5	0 (0)	1 (0.3)	
*Self-care*			
Level 1	121 (100)	377 (97)	0.63
Level 2	0 (0)	7 (2)	
Level 3	0 (0)	2 (1)	
Level 4	0 (0)	0 (0)	
Level 5	0 (0)	1 (0.3)	
*Usual activity*			
Level 1	119 (98)	367 (95)	0.39
Level 2	2 (2)	15 (4)	
Level 3	0 (0)	4 (1)	
Level 4	0 (0)	0 (0)	
Level 5	0 (0)	1 (0.3)	
*Pain/discomfort*			
Level 1	80 (66)	236 (61)	0.86
Level 2	34 (28)	126 (33)	
Level 3	6 (5)	21 (5)	
Level 4	1 (1)	3 (1)	
Level 5	0 (0)	1 (0.3)	
*Anxiety/depression*			
Level 1	85 (70)	276 (71)	0.94
Level 2	34 (28)	104 (27)	
Level 3	2 (2)	6 (2)	
Level 4	0 (0)	1 (0.3)	
Level 5	0 (0)	0 (0)	

P4P^a^: pay-for-performance; *P* value^b^: The *P* values were derived from the chi-square test

The Kolmogorov–Smirnov test revealed skewed distributions of the EQ5D utility scores and VAS scores. Accordingly, mean (standard deviation, SD) and median (interquartile range, IQR) scores are presented. The mean (SD) EQ-5D index and EQ-VAS scores were 0.93 (0.12) and 75.1 (13.8), and the median (IQR) values were 1 (0.108) and 80 (15), respectively (data not shown). Table 3 shows the effect of the hepatitis P4P program based on different clinical variables using univariable analysis. The EQ-VAS score showed significant improvement in all patients after participating in the hepatitis P4P program (*P* = 0.01). After performing a subgroup analysis according to the clinical variables, the differences were also significant among the patients with comorbidities, patients with hepatitis-related complications, patients without admission within 1 year (for hepatitis-related events), patients with or without emergency department visits within one year and patients without admissions within one year (for hepatitis-related events), and other hepatitis (hepatitis C and hepatitis B/C coinfection). However, hepatitis P4P did not substantially change patients' EQ-5D index, regardless of subgroup.

**Table 3:** Effect of the hepatitis pay-for-performance program on EQ5-5D-5L, according to different clinical variables

	EQ-5D index			EQ-VAS score		
	P4P^a^	Non- P4P^a^	*P* value	P4P^a^	Non- P4P^a^	*P* value
All	0.937 (0.085)	0.925 (0.132)	0.32	78.29 (12.04)	74.07 (14.21)	0.01
Any comorbidity	0.933 (0.089)	0.913 (0.148)	0.30	78.31 (12.56)	72.22 (14.33)	0.002
No	0.943 (0.080)	0.944 (0.094)	0.92	78.26 (11.48)	77.29 (13.45)	0.64
Any Complication	0.945 (0.089)	0.898 (0.238)	0.36	81.30 (11.00)	71.51 (14.83)	0.01
No	0.935 (0.084)	0.930 (0.098)	0.61	77.58 (12.22)	74.56 (14.06)	0.06
Admission within one year	0.914 (0.149)	0.790 (0.386)	0.60	81.76 (02.89)	69.90 (19.32)	0.31
No	0.938 (0.083)	0.932 (0.097)	0.55	78.20 (12.18)	74.29 (13.88)	0.01
Emergency department visit within one year	0.936 (0.129)	0.769 (0.416)	0.45	80.00 (04.08)	66.65 (19.49)	0.02
No	0.937 (0.084)	0.932 (0.097)	0.58	78.23 (12.23)	74.41 (13.86)	0.01
*Viral hepatitis*						
Hepatitis B	0.942 (0.083)	0.932 (0.094)	0.33	78.05 (12.54)	74.94 (13.75)	0.05
Other hepatitis^b^	0.908 (0.017)	0.910 (0.124)	0.76	78.20 (10.07)	71.78 (15.20)	0.004

Values of scores are presented as the mean (SD; standard deviation); P4P^a^: pay-for-performance; Other hepatitis^b^: hepatitis C and hepatitis B/C coinfection

Table 4 shows the multiple linear regression results obtained upon applying PSW adjustment in addition to adjusting for the other covariables. The regression analysis showed no multicollinearity between independent variables. In this model, P4P participation was associated with higher EQ-VAS scores; however, the difference was nonsignificant (β = 2.25; 95% CI = − 0.025–4.534; *P* = 0.05). EQ-VAS scores of the patients with income levels ranging from US$2,001 to $3,333 per month (β = 6.813; 95% CI = 2.683–10.943; *P* = 0.001) and greater than US$3,333 per month (β = 7.997; 95% CI = 1.669–14.325; *P* = 0.01) were higher than those of patients with income levels less than US$1,000. Married patients exhibited higher EQ-VAS score than unmarried patients (β = − 5.290; 95% CI = − 9.450 to − 1.131; *P* = 0.01) and patients with other marital statuses, including separated and divorced patients (β = − 7.900; 95% CI = − 12.430 to − 3.364; *P* < 0.001). Association between P4P participation and the EQ-5D index was nonsignificant in the regression model with PSW adjustment in addition to the other covariables. The EQ-5D index values are significantly associated with marital status, comorbidity, and type of hepatitis patient infected. Regarding marital status, widowed patients (β = − 0.059; 95% CI = − 0.101 to − 0.016; *P* = 0.01) and patients with other marital statuses (β = − 0.054; 95% CI = − 0.090 to − 0.018; *P* = 0.003) had lower EQ-5D index values than married patients. Patients who had any one of the comorbidities had lower index scores than those without (β = − 0.025; 95% CI = − 0.044 to − 0.005; *P* = 0.01). Patients infected with hepatitis B (β = 0.024; 95% CI = 0.002–0.046; *P* = 0.03) exhibited higher EQ-5D index scores than patients infected with hepatitis C and coinfected with hepatitis B/C. The R squared values of the regression models for the EQ-5D index and EQ-VAS scores were 0.14 and 0.10, respectively.

**Table 4:** Estimated propensity score weighting adjustment coefficients for EQ-5D in the multiple ordinary least squares regression model

	EQ-5D index			EQ-VAS score			VIF^e^
	β value	*P*-value	95% CI^b^	β value	*P*-value	95% CI^b^	
P4P^a^ participation	< − 0.001	0.68	− 0.022 to 0.014	2.25	0.05	− 0.03 to 4.53	1.095
Male (Ref: female)	0.017	0.11	− 0.004 to 0.038	− 0.49	0.71	− 3.07 to 2.09	1.207
Age	< 0.001	0.74	< − 0.001 to 0.001	− 0.02	0.73	− 1.61 to 0.11	1.824
*Income (Ref: income* < *US$ 1000)*							
U.S.$1000–$2000	0.018	0.12	− 0.005 to 0.042	2.08	0.16	− 0.84 to 5.00	1.447
U.S.$2001–$3333	0.037	0.03	0.004 to 0.070	6.81	0.001	2.68 to 10.94	1.370
U.S.$ > 3333	0.037	0.15	− 0.014 to 0.088	8.00	0.01	1.67 to 14.33	1.263
*Education (Ref: Below elementary)*							
Junior high	0.036	0.02	0.005 to 0.067	2.52	0.20	− 1.32 to 6.35	1.693
College	0.009	0.62	− 0.028 to 0.046	2.16	0.37	− 2.49 to 6.75	1.718
Senior high	0.024	0.08	− 0.033 to 0.052	1.82	0.30	− 1.63 to 5.28	2.074
Bachelor	0.003	0.90	− 0.036 to 0.041	2.27	0.36	− 2.58 to 7.13	2.132
Master & Doctorate	− 0.009	0.73	− 0.061 to 0.043	3.31	0.49	− 4.23 to 8.76	1.499
*Marital status (Ref: married)*							
Unmarried	− 0.028	0.10	− 0.061 to 0.005	− 5.29	0.01	− 9.45 to − 1.13	1.278
Widow	− 0.059	0.01	− 0.101 to − 0.016	0.31	0.91	− 4.99 to 5.61	1.112
Other^c^	− 0.054	0.003	− 0.090 to − 0.018	− 7.90	< 0.001	− 12.43 to − 3.36	1.113
Any comorbidity	− 0.025	0.01	− 0.044 to − 0.005	− 1.04	0.41	− 3.50 to 1.42	1.120
Any complication	0.007	0.60	− 0.019 to 0.033	1.13	0.49	− 2.10 to 5.61	1.119
Admission within one year	− 0.067	0.10	− 0.145 to 0.012	3.42	0.49	− 6.38 to 13.23	2.437
Emergency department visit within one year	− 0.052	0.20	− 0.133 to 0.028	− 6.12	0.23	− 16.18 to 3.94	2.459
Hepatitis B (Other hepatitis^d^)	0.024	0.03	0.002 to 0.046	− 0.38	0.78	− 3.11 to 2.34	1.129
R-square	0.14			0.10			

P4P^a^: pay-for-performance; CI^b^: confidence interval; Other^c^: separated and divorced status; Other hepatitis^d^: hepatitis C and hepatitis B/C coinfection; VIF^e^: variance inflation factor for ordinary least squares regression

Table 5 shows the regression analysis of the EQ-5D disutility scores obtained using the two-part model and the results of the EQ-VAS scores obtained using GLM Poisson with PSW adjustment and adjusting for the other covariables. P4P participation was significantly associated with higher EQ-VAS scores (mean ratio = 1.029; 95% CI = 1.014–1.044; *P* < 0.001). The monthly income, education level, and marital status of the hepatitis patients also were significantly associated with the EQ-VAS score. Regarding clinical considerations, patients who had visited the emergency department within one year for hepatitis-related events showed lower EQ-VAS scores (mean ratio = 0.935; 95% CI = 0.877–0.997; *P* = 0.04). P4P participation was not significantly associated with the EQ-5D disutility score in the two-part model after applying PSW adjustment and adjusting for the other covariables (β = − 0.12; 95% CI = − 0.28–0.04; *P* = 0.14). The regression results revealed that the disutility score was associated with patients’ education level (junior high β = − 0.30; 95% CI = − 0.53 to − 0.06 *P* = 0.01; senior high β = − 0.30; 95% CI = − 0.52 to − 0.09 *P* = 0.01; reference: below elementary). The other factors did not show a significant effect in the analysis.

**Table 5:** Estimated propensity score weighting adjustment coefficients for EQ-5D in the two-part model and generalized linear model

	Disutility score (EQ-5D index)^e^			EQ-VAS score		
	Coefficient	*P*-value	95% CI^b^	Mean ratio^f^	*P*-value	95% CI^b^
P4P^a^ participation	− 0.121	0.14	− 0.283 to 0.041	1.03	< 0.001	1.01 to 1.04
Male (Ref: female)	− 0.010	0.90	− 0.168 to 0.148	0.99	0.37	0.98 to 1.01
Age	0.007	0.28	− 0.005 to 0.020	1.00	0.60	0.99 to 1.00
*Income (Ref: income* < *US$ 1000)*						
U.S.$1,000–$2,000	− 0.009	0.94	− 0.245 to 0.228	1.03	0.002	1.01 to 1.05
U.S.$2,001–$3,333	− 0.115	0.50	− 0.449 to 0.219	1.09	< 0.001	1.06 to 1.12
U.S.$ > 3,333	− 0.159	0.29	− 0.451 to 0.133	1.11	< 0.001	1.07 to 1.15
*Education (Ref: Below elementary)*						
Junior high	− 0.296	0.01	− 0.533 to − 0.059	1.03	0.01	1.00 to 1.06
College	− 0.016	0.92	− 0.340 to 0.308	1.62	0.06	0.99 to 1.06
Senior high	− 0.305	0.01	− 0.522 to − 0.088	1.02	0.03	1.00 to 1.04
Bachelor	− 0.108	0.59	− 0.501 to 0.285	1.03	0.048	1.00 to 1.06
Master & Doctorate	− 0.111	0.60	− 0.526 to 0.303	1.03	0.13	0.99 to 1.07
*Marital status (Ref: married)*						
Unmarried	0.296	0.09	− 0.019 to 0.612	0.99	< 0.001	0.91 to 0.96
Widow	0.322	0.22	− 0.052 to 0.697	1.02	0.26	0.99 to 1.05
Other^c^	0.210	0.10	− 0.127 to 0.547	0.90	< 0.001	0.87 to 0.93
Any comorbidity	0.168	0.10	− 0.033 to 0.369	0.99	0.09	0.97 to 1.00
Any complication	− 0.105	0.39	− 0.346 to 0.136	1.02	0.10	0.99 to 1.04
Admission within one year	0.118	0.76	− 0.655 to 0.892	1.06	0.07	0.99 to 1.13
Emergency department visit within one year	0.516	0.26	− 0.386 to 1.419	0.94	0.04	0.88 to 1.00
Hepatitis B (Ref: Others hepatitis^d^)	− 0.025	0.80	− 0.214 to 0.165	1.18	0.37	0.98 to 1.01

P4P^a^: pay-for-performance; CI^b^: confidence interval; Other^c^: separated and divorced status; Other hepatitis^d^: hepatitis C and hepatitis B/C coinfection; Disutility score (EQ-5D index)^e^: two-part model on disutility score; Mean ratio^f^: Generalized linear model for the natural log of the mean reported EQ-VAS score

## Discussion

To the best of our knowledge, this is the first study to investigate the effect of hepatitis P4P on HRQoL. Our results demonstrate that hepatitis P4P was associated with significantly higher EQ-VAS scores in chronic viral hepatitis patients. However, such interventions were not significantly associated with improvements in the EQ-5D index values.

Previous HRQoL studies that employed the EQ-5D have shown that the mean EQ-5D index scores vary from 0.37 to 0.93, and the range of the mean EQ-VAS scores has been reported to be between 57 and 88 [24, 36, 52–58]. Patients who have higher incomes, are married, and have higher education have higher EQ-VAS scores [57, 58], similar to our results. In our study, the mean EQ-5D index and EQ-VAS scores were 0.92 and 75.4, respectively, which is within the range of previous studies and similar to the EQ-5D results obtained from hepatitis patients in Korea (average EQ-5D index/EQ-VAS = 0.93/70.2) [57] with a similar socioeconomic profile and healthcare environment. Our study showed that the P4P and non-P4P groups were dissimilar in only two demographic characteristics: income and education levels; therefore, heterogeneity in the P4P and non-P4P groups was slightly high. However, the P4P and non-P4P groups were similar in clinical characteristics. In a previous study [59], patients with more severe disease or comorbidities were more prone to participate in a P4P program; this was not the case in the present study because the two groups investigated were identical in terms of comorbidities and disease severity. We also applied a rigorous PSW methodology to compensate for the different distributions within the two groups (income and education), which minimized selection bias. In addition, a previous study using a nationwide claim database exhibited a 59% distribution of male hepatitis patients and an average age of approximately 52 [20]. In the present study, the distribution of male hepatitis patients was 61%, and the average age was 58, which is slightly higher than the average age of the national population. We believe our sample more accurately represents (external validity) Taiwan. With regard to modeling, we discuss the regression model structure and explanatory power (R-square) below. It may be that OLS regression is a poor regression structure due to skewness or ceiling effects associated with the EQ-5D index and EQ-VAS scores [47, 48]. To adjust for these problems, we applied a two-part model and GLM, and the results demonstrated an association between hepatitis P4P and the EQ-VAS score but not the EQ-5D index. This is in contrast to the OLS results, which indicate that P4P is not associated with the EQ-VAS score. The OLS regression results showed that P4P participation did not significantly increase the EQ-VAS (2.25) score; however, the significance observed was borderline (*P* = 0.05). We do not clearly understand whether hepatitis P4P participation achieves a minimal clinically important difference (MCID) or responsiveness if 2.25 is considered significant. Other studies that are not related to hepatitis care have shown an MCID for EQ-VAS scores ranging from 0.39 to 10.82 [40, 60, 61]. Further study should investigate the effect of hepatitis P4P intervention on the MCID. Regarding model explanatory power, we tried our best to include possible confounders, such as clinical or socioeconomic factors. The R-square values for HRQoL reported in other hepatitis patient studies range from 0.05 to 0.47 [36, 52, 62, 63]. We cannot rule out the possibility that other variables affecting the EQ-5D index and EQ-VAS scores were not considered in our analysis (omitting variable bias). Further research should explore other factors that may be associated with the EQ-5D index and EQ-VAS score.

The association between hepatitis P4P and EQ-VAS health scores may be due to regular and ongoing examinations, continuity of care, physician trust, and control of comorbidity. With regard to the first, hepatitis P4P in Taiwan is characterized by regular and ongoing essential examinations, as studies have shown that examinations can promote patients’ overall health. For example, the United Kingdom (U.K.) P4P programs that target general practitioners include measures related to examinations in different fields, and studies have shown that in the U.K., P4P affects patient outcomes [64] but not quality of life [65]. Regarding continuity of care, patients with chronic diseases who have better continuity of care have been known to have better clinical outcomes [66] and decreased mortality [67]. In addition, studies have shown that continuity of care is associated with better HRQoL in patients with diabetes [68], hypertension [69], and multiple chronic medical conditions [70]. Third, regarding the physician trust issue, compared to patients with irregular or few visits, patients with regular and continuous visits should have a higher chance of building a relationship of trust with their physicians. Consequently, patients with regular and continuous visits are more willing to self-manage their overall health [71]. Fourth, regarding well-treated comorbidities, previous research has shown that patients with more comorbidities are more likely to visit physicians, and those physicians are more likely to administer highly recommended care [72–74]. In other words, patients with regular visits are more likely to be transferred to uptake other highly recommended care practices that enable better outcomes than non-P4P participants.

The following considerations may help explain the ineffectiveness of hepatitis P4P on the EQ-5D index. Previous studies have shown that hepatitis P4P, which purely targets increased regular examinations, does not sufficiently reduce incidences of admission or cirrhosis [20], which may be one reason for the indifference in QoL observed in the P4P versus control groups. Another study with more extensive intervention, reported by Chao et al. [75], demonstrates that after one year of comprehensive care, including government support, technical guidance, standardized medical care, and community involvement, hepatitis B patients significantly improved in terms of HRQoL, perhaps implying that not only regular examinations but also multiple-dimensional interventions are needed to improve EQ-5D index effects. It is also noteworthy that the study design did not allow for before/after comparisons. In addition, we expect to observe no large HRQoL differences between P4P participants and nonparticipants when complications and symptoms are rare. Finally, the hepatitis P4P program focused on preventive liver examination but not antiviral treatment. Hence, differences in the P4P group and non-P4P group were small because treatment has already been shown to be adequate and effective in Taiwan [76].

It is important to note the limitations of this study. First, the cross-sectional study does not permit analysis of the long-term relationship between P4P and QoL. Future studies should examine the abovementioned effect in a longitudinal manner. Second, regarding the validity and reliability of the EQ-5D-5L values obtained using Taiwanese value sets, few hepatitis patient studies exist in this regard because the Taiwanese value set has only been proposed in recent years [45]. In one study, the EQ-5D-5L results obtained using U.K. and Japanese value sets have shown good concurrent, discriminant, and convergent validity via comparison with SF-36 on a national representative sample in Taiwan [39]. In addition, one early study demonstrated good internal reliability for the Taiwanese EQ-5D-5L (Cronbach’s α: 0.78) for patients with stroke [40]. Our study derived similar results when Japanese EQ-5D-5L value sets were used, indicating that the utility score is valid when applied to patients with hepatitis (data not shown). Further studies should investigate validity and reliability issues associated with the Taiwanese EQ-5D-5L for patients with hepatitis by using a recently proposed value set. Third, we did not adopt questionnaires regarding disease-specific quality of life, such as the chronic liver disease questionnaire (CLDQ), because of the following advantages associated with the EQ-5D, which is a simpler questionnaire [35, 36] with fair convergent validity compared to the CLDQ (correlation is approximately 50%) [77]. In addition, the EQ-5D has been broadly used as an outcome measure to evaluate intervention outcomes for patients with hepatitis [53, 54, 58, 78–81]. Future studies could also use the CLDQ to evaluate the effectiveness of hepatitis P4P on HRQoL and verify the results derived from our study. Fourth, we tried our best to include possible confounders, such as clinical and socioeconomic factors. The R-square values for the EQ-5D index and EQ-VAS score reported in other hepatitis patient studies were not lower than our values [36, 52, 62, 63]; hence, we must consider the likely possibility that other variables (i.e., health literacy or health behavior) affecting the EQ-5D index and EQ-VAS values are missing in our analysis. Finally, our results may be confounded by concurrent antiviral treatment [20], which may be associated with QoL. However, we think this effect is probably small because we collected data at only four hospitals. According to a Ministry of Health and Welfare report [30], in 2016, approximately 62,000 hepatitis B patients underwent antiviral therapy, 2.5% of all hepatitis B patients in Taiwan and approximately 5000 hepatitis C patients received antiviral treatment, accounting for approximately 1.3% of all hepatitis C patients. Therefore, the confounding effect of antiviral therapy on our report is likely limited. However, we cannot rule out the possibility that confounding effect occurs if data are derived from the NHIA’s databases.

## Conclusion

Patients with hepatitis partially benefited from receiving hepatitis P4P in Taiwan, featuring regular ongoing examinations, as evidenced by enhancement of their EQ-VAS scores but not their EQ-5D indexes.

## Data Availability

The datasets during and/or analyzed during the current study available from the corresponding author on reasonable request.

## Abbreviations

HRQoL: Health-related quality of life
P4P: Pay-for-performance
EQ-5D: EuroQol-5 dimension
EQ-5D-5L: Five-level version EQ-5D
EQ-VAS: EuroQoL visual analog scale
GLM: Generalized linear model
PSW: Propensity score weighting
HBV: Hepatitis B
HCV: Hepatitis C
HCC: Hepatocellular carcinoma
GOT: Glutamic oxaloacetic transaminase
GPT: Glutamic pyruvic transaminase
NHIA: National health insurance administration
PROs: Patient-reported outcomes
QoL: Quality of life
IRB: Institutional review board
VIF: Variance inflation factor
OLS: Ordinary least squares
IPTW: Inverse probability treatment weight
SMRW: Standardized mortality ratio weight
SE: Standard deviation
IQR: Interquartile range
MCID: Minimal clinically important difference
CLDQ: Chronic liver disease questionnaire
CI: Confidence interval
U.K.: United Kingdom
WHO: World Health Organization
